# Liver Adiposity and Metabolic Profile in Individuals with Chronic Spinal Cord Injury

**DOI:** 10.1155/2017/1364818

**Published:** 2017-08-30

**Authors:** Kathleen C. Rankin, Laura C. O'Brien, Liron Segal, M. Rehan Khan, Ashraf S. Gorgey

**Affiliations:** ^1^Spinal Cord Injury and Disorders, Hunter Holmes McGuire VA Medical Center, Richmond, VA, USA; ^2^Department of Physiology and Biophysics, Virginia Commonwealth University, Richmond, VA, USA; ^3^Department of Radiology, Virginia Commonwealth University, Richmond, VA, USA; ^4^Department of Physical Medicine and Rehabilitation, Virginia Commonwealth University, Richmond, VA, USA

## Abstract

**Purpose:**

To quantify liver adiposity using magnetic resonance imaging (MRI) and to determine its association with metabolic profile in men with spinal cord injury (SCI).

**Materials and Methods:**

MRI analysis of liver adiposity by fat signal fraction (FSF) and visceral adipose tissue (VAT) was completed on twenty participants. Intravenous glucose tolerance test was conducted to measure glucose effectiveness (*S*_g_) and insulin sensitivity (*S*_i_). Lipid panel, fasting glucose, glycated hemoglobin (HbA1c), and inflammatory cytokines were also analyzed.

**Results:**

Average hepatic FSF was 3.7% ± 2.1. FSF was positively related to TG, non-HDL-C, fasting glucose, HbA1c, VAT, and tumor necrosis factor alpha (TNF-*α*). FSF was negatively related to *S*_i_ and testosterone. FSF was positively related to VAT (*r* = 0.48, *p* = 0.032) and TNF-*α* (*r* = 0.51, *p* = 0.016) independent of age, level of injury (LOI), and time since injury (TSI). The associations between FSF and metabolic profile were independent of VAT.

**Conclusions:**

MRI noninvasively estimated hepatic adiposity in men with chronic SCI. FSF was associated with dysfunction in metabolic profile, central adiposity, and inflammation. Importantly, liver adiposity influenced metabolic profile independently of VAT. These findings highlight the significance of quantifying liver adiposity after SCI to attenuate the development of metabolic disorders.

## 1. Introduction

Metabolic dysfunction following spinal cord injury (SCI) is characterized by a high prevalence of lipid disorders, impaired glucose tolerance, and insulin resistance [1–3]. Metabolic dysfunction is linked to decreased muscle mass, increased fat mass, and low anabolic hormones [1–10]. For instance, 76% of individuals with SCI were shown to have dyslipidemia [4] with another study showing that 50% of individuals with paraplegia and 62% of those with tetraplegia had impaired glucose tolerance [5]. Two-thirds of individuals with SCI are overweight or obese regardless of a body mass index (BMI) below 30 kg/m^2^, because BMI underestimates the percentage of body fat in this population [3, 6–8]. Furthermore, persons with SCI experience decreased anabolic hormone levels, which is associated with abnormal lipid and metabolic profiles. A previous study showed that 43% of men with chronic SCI had low testosterone [9]. Therefore, studying factors that may explain the high prevalence of metabolic disease is clinically relevant to this population.

The associations between increased adipose tissue and metabolic disorders are well established in the SCI population. A previous study showed that an increase in trunk and leg fat mass was related to deterioration of lipid and metabolic profile [10]. This confirmed earlier reports that showed that intramuscular fat contributed to insulin resistance [11] and predicted plasma glucose and type II diabetes in individuals with SCI [12]. Furthermore, visceral adipose tissue (VAT) is tightly linked to abnormal lipid and carbohydrate profiles after SCI [10, 13]. Gorgey et al. showed that VAT was positively related to fasting plasma glucose and total cholesterol [10]. It appears that regional ectopic adiposity has detrimental effects on metabolic profile, but VAT or regional adiposity did not entirely explain the variance in metabolic dysfunction after SCI [10]. This may be explained by the use of different imaging techniques or failure to account for all depots of ectopic adiposity. Therefore, accounting for different depots of ectopic adiposity (VAT versus hepatic adiposity) may provide insights on a range of metabolic disorders after SCI.

Magnetic resonance imaging (MRI) technology is a noninvasive technique used to quantify adipose tissue depots such as VAT, subcutaneous adipose tissue (SAT), intramuscular fat, and most recently bone marrow and liver adiposity [14, 15]. This is extremely advantageous in understanding the complexity of metabolic profile, since it remains unclear how different adipose tissue depots contribute to metabolic dysfunction after SCI. Studies have demonstrated that liver adipose tissue is positively related to VAT and has also been linked to the development of type II diabetes mellitus and insulin resistance [16, 17]. Previous studies have speculated that the release of inflammatory cytokines from ectopic adipose tissue contributes to a state of chronic inflammation and progression of metabolic disease [18]. Therefore, understanding the influence of various adipose tissue depots on metabolic health is critical for elucidating the mechanisms underlying the progression of metabolic disease. This may allow for early interventions to prevent adverse health consequences after SCI.

Abnormal amounts of liver adipose tissue that is not explained by excessive alcohol consumption is known as nonalcoholic fatty liver disease (NAFLD)—a term that encompasses steatosis, steatohepatitis, and cirrhosis [19]. Steatosis is the simplest stage of NAFLD and is characterized as >5% fatty-infiltrated hepatocytes [20]. Steatosis has a prevalence of ~30% in the general population [21] and ~90% in the obese population [22]. Recent studies have shown that NAFLD is strongly associated with increased incidence of metabolic syndrome, type II diabetes [23–25], and increased inflammation [26–28]. Approximately 20%–50% of persons with chronic SCI had abnormal fatty-infiltration of the liver determined by ultrasound [29, 30] and animal studies of acute SCI have documented increased liver inflammation [31, 32].

Liver fat can be quantified by liver biopsy, hydrogen magnetic resonance spectroscopy (^1^H-MRS), MRI, computed tomography (CT), and ultrasound [33]. For years, the gold standard for diagnosing NAFLD has been a liver biopsy. However, the reliability of this invasive procedure has been debated as it samples only ~1/50,000 of the total liver mass [20]. MRI provides a simple tool that allows for the detection of liver fat. Quantifying liver adiposity by MRI relies on the signal intensity of the in-phase (IP) and out-of-phase (OP) images, where the IP image represents the signal intensity of the sum of water and fat and the OP image represents the difference of water and fat [34]. Fat signal fraction (FSF) can be calculated from these values and has been shown to be an accurate and reproducible technique to measure liver fat content [33, 35, 36]. Previous studies have shown strong correlations between FSF and histological liver fat content [37, 38]. Increased FSF was observed in obese individuals and decreased after weight loss [35, 37, 39]. Another study revealed that FSF was associated with deterioration in lipid profile in men and women [40] and central obesity in healthy men [38].

The purpose of the current study was to quantify liver adiposity in a chronic SCI population using FSF determined by MRI. We furthermore aimed to investigate the relationships between FSF and both metabolic and lipid profiles. The hypothesis was that FSF would be positively related to serum triglycerides (TG), free-fatty acids (FFA), and VAT and negatively related to insulin sensitivity. It was of additional interest to investigate the association between FSF and inflammatory biomarkers. We are unaware of any study that has investigated the association between liver adiposity and metabolic dysfunction independent of VAT in persons with SCI.

## 2. Methods

This study was given ethical approval by the McGuire VA Medical Center institutional review board. All procedures were in accordance with the Helsinki Declaration of 1964 ethical standards. Written informed consent was obtained from each participant as part of a clinical trial (NCT01652040). Data presented was obtained at baseline, prior to the intervention, and the study protocol has been published separately [41].

### 2.1. Participants

Twenty-two men ages 18–50 with motor complete SCI were recruited for the study. Participant demographics are summarized in Table 1. Each of the participants' time since injury (TSI) was greater than one year. Participants were classified according to the American Spinal Injury Association Impairment Scale (AIS) as A or B. Levels of injury ranged from T11 to C5. Exclusion criteria included known cardiovascular disease, uncontrolled type II diabetes mellitus, or pressure sores stage II or greater. All participants completed a physical and medical examination to determine study eligibility. This examination included a neurological assessment according to the International Standards for Neurological Classification of SCI (ISNCSCI), including the American Spinal Injury Association (ASIA) Impairment Scale (AIS). Inclusion criteria included men with motor complete SCI level C5-L2 greater than one year after injury, ages 18–50.

### 2.2. Metabolic Profile

Metabolic profile was assessed as previously described [41, 42]. Blood samples were taken every 2 minutes before and 2-3 minutes after glucose injection (0.3 g/kg) for 30 minutes, followed by sampling every 5, 10, 20, and 30 minutes ending at 180 minutes. Twenty minutes after glucose injection insulin (0.02 U/kg) was injected. Insulin sensitivity (*S*_i_) and glucose effectiveness (*S*_g_) were determined using MinMod software (MinMod Inc., Pasadena, CA) [43]. Three values for *S*_i_ were excluded due to measurement error. Fasted lipid profile and total testosterone levels were measured prior to glucose injection. Non-high-density lipoprotein cholesterol (non-HDL-C) is representative of low-density lipoprotein cholesterol (LDL-C) and very low LDL-C (VLDL-C). One participant's values for both plasma glucose and glycated hemoglobin (HbA1c) were inaccessible due to a technical lab error and one participant did not undergo any metabolic profile measurements.

### 2.3. Inflammatory Cytokines

Inflammatory cytokines were measured from fasting serum samples with a specific ELISA for human tumor necrosis factor alpha (TNF-*α*) and for human interleukin-6 (IL-6) according to the manufacturer's instructions (ALPCO). Data are expressed in picograms per milliliter.

### 2.4. Magnetic Resonance Imaging (MRI)

IP and OP images were obtained with a 1.5 T scanner using an axial T1-weighted fast spin-echo image acquisition (General Electric Signa scanner, Milwaukee, WI, USA). Transverse slices were taken from the xiphoid process to the femoral heads. The legs and knees were strapped to prevent muscle spasms. Participants were asked to hold their breath to reduce artifact [8]. Parameters were as follows: repetition time, 140 ms; echo time, 4.3 ms (IP), 2 ms (OP); flip angle, 80°; field of view, 42 cm; slice thickness, 0.8 cm; interslice space, 1.2 cm [8]. MRI data was not available for one participant, so only metabolic and lipid data were analyzed for this individual.

Images were sequenced anatomically and analyzed using specialized imaging software for MRI analysis (Win Vessel 2, Ronald Meyer, MSU, MI, USA). Calculation of VAT and SAT has been detailed previously [10]. Briefly, an experienced technician manually traced regions of interest using segmentation and signal intensity to identify the fat and nonfat tissue. Trunk cross-sectional area (CSA) refers to SAT and VAT, in addition to other nonfat compartments such as bone and organs.

### 2.5. Liver Cross-Sectional Area (CSA) and Fat Signal Fraction (FSF)

**Figure 1:**
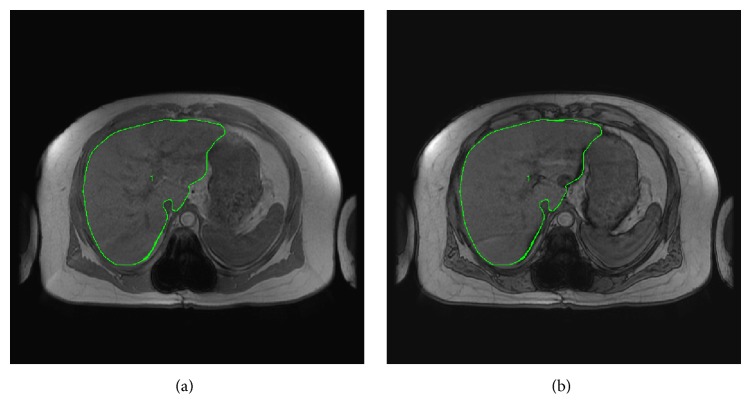
MRI images were used to trace the liver using the in-phase (IP) image (a) and its identical out-phase (OP) image (b) for calculation of the hepatic fat signal fraction (FSF).

Three IP images were used from the start of the anatomical sequence to analyze the liver at its largest size. Each image's IP was matched to its identical OP and all three coupled images were matched among each participant as best as possible. The technician traced around the whole liver in the IP image excluding large veins and arteries (Figure 1(a)). This trace was copied to its identical OP image (Figure 1(b)). Signal intensity and CSA were recorded for both IP and OP images. Liver CSA was measured by multiplying the total number of pixels by the pixel size (field of view/matrix size)^2^. The following equation [44] was used to calculate the liver FSF:

(1) $$\begin{array}{c} FSF=Abs\left[\;\frac{\left({SI}_{IP}-{SI}_{OP}\right)}{2*{SI}_{IP}}\;\right], \end{array}$$

where Abs is absolute value, SI_IP_ is the signal intensity of the liver in the IP image, and SI_OP_ is the signal intensity of the liver in the OP image. Two participants were excluded from analysis due to poor image quality. FSF-VAT index was calculated as (FSF + VAT)/SAT.

### 2.6. Statistics

IBM-SPSS version 23 (Armonk, NY) was used for all statistical analyses. Differences between individuals with paraplegia (T4–T11) and tetraplegia (C5–C7) were analyzed using independent *t*-tests. Bivariate Pearson correlations were used to determine the relationship between hepatic FSF and lipid and metabolic profile variables. Age, level of injury (LOI), and TSI were accounted for by partial correlations. Partial correlations were also used to account for VAT and inflammatory cytokines in the relationships between FSF and metabolic profile. Statistical significance was determined at *p* < 0.05. Data presented is mean ± standard deviation (SD).

## 3. Results

### 3.1. Participant Characteristics

The physical characteristics of study participants are presented in Table 1. There were no significant differences between individuals with tetraplegia and paraplegia or between ethnic groups. Lipid profile, metabolic profile, adipose tissue, inflammation, and liver measurements are presented in Table 2.

Non-HDL-C and total cholesterol : HDL-C were significantly greater in individuals with tetraplegia compared to those with paraplegia. There was a trend for higher fasting glucose and HbA1c and lower *S*_i_ in individuals with tetraplegia compared to those with paraplegia. Individuals with tetraplegia had 36% less testosterone compared to those with paraplegia. No significant differences were observed in inflammatory cytokines, trunk CSA, SAT, or VAT.

### 3.2. Liver Cross-Sectional Area (CSA) and Fat Signal Fraction (FSF)

Liver CSA was not significantly different between individuals with tetraplegia and those with paraplegia (Table 2). Thirty percent of participants had an FSF greater than 5%, with a range of 0.5–8.1%. Average hepatic FSF was 3.7 ± 2.1%, with higher values in individuals with tetraplegia (5.3 ± 2.1%) compared to individuals with paraplegia (2.9 ± 1.6%; *p* = 0.008).

### 3.3. Relationships among FSF and Lipid and Metabolic Profiles

**Figure 2:**
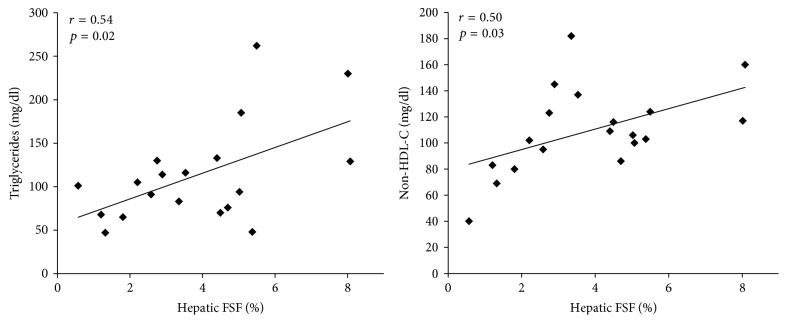
Relationships between hepatic fat signal fraction (FSF) and lipid profile. TG, triglycerides and Non-HDL-C, high-density lipoprotein cholesterol.

**Figure 3:**
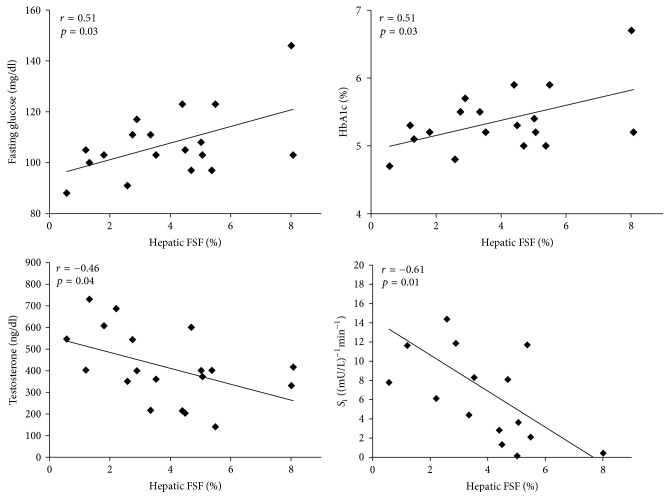
Relationships between hepatic fat signal fraction (FSF) and metabolic profile. Fasting glucose, HbA1c, glycated hemoglobin, testosterone, and *S*_i_, insulin sensitivity.

The relationships between FSF and lipid profile are shown in Figure 2. FSF was positively related to TG (*r* = 0.54, *p* = 0.017) and non-HDL-C (*r* = 0.50, *p* = 0.028). There was no relationship between FSF and total cholesterol, LDL-C, HDL-C, total cholesterol : HDL ratio, or FFA. The relationships between FSF and metabolic profile are shown in Figure 3. There were positive relationships between FSF, fasting glucose, and HbA1c (*r* = 0.51, *p* = 0.029 and *r* = 0.51, *p* = 0.032, resp.). FSF was negatively related to *S*_i_ (*r* = −0.61, *p* = 0.013) and testosterone (*r* = −0.47, *p* = 0.044). There was no relationship between FSF and glucose effectiveness (*S*_g_). When ectopic adipose tissue (FSF + VAT) was normalized to SAT (FSF-VAT index), these relationships were no longer significant. There were, however, trends between the FSF-VAT index and fasting glucose, HbA1c, and FFA (*r* = 0.43  *p* = 0.073, *r* = 0.44  *p* = 0.069, and *r* = 0.49  *p* = 0.067, resp.).

### 3.4. Relationships among FSF, Trunk Adipose Tissue, and Inflammation

**Figure 4:**
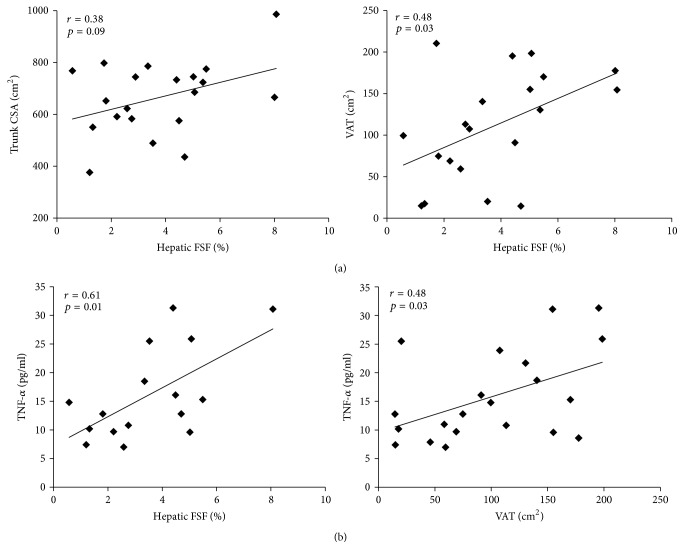
Relationships between hepatic fat signal fraction (FSF), trunk cross-sectional area (CSA), and visceral adipose tissue (VAT; (a)) and inflammation and ectopic adipose tissue ((b); hepatic FSF or VAT). TNF*α*, tumor necrosis factor alpha.

The relationships between FSF, trunk CSA, and VAT are shown in Figure 4(a). There was a trend between FSF and trunk CSA (*r* = 0.38, *p* = 0.094). There was a significant positive relationship between FSF and VAT (*r* = 0.48, *p* = 0.032), but no significant relationship with SAT.  Figure 4(b) shows the relationships between ectopic adipose tissue and inflammation. FSF was strongly related to the TNF-*α* (*r* = 0.62, *p* = 0.012) but not IL-6. TNF-*α* was also positively related to VAT (*r* = 0.48, *p* = 0.028). Furthermore, the relationships between the FSF-VAT index and inflammatory cytokines were not significant.

### 3.5. Relationships Independent of Age, Level of Injury (LOI), and Time since Injury (TSI)

Table 3 shows the relationships between FSF and metabolic and lipid profiles after accounting for age, LOI, and TSI. The relationships between FSF and TG, *S*_i_, VAT, and TNF-*α* remained significant after accounting for age and TSI. Non-HDL-C remained significant after accounting for age but was no longer significant after considering TSI. Trends were seen between FSF and fasting glucose (*p* = 0.053), HbA1c (*p* = 0.057), and testosterone (*p* = 0.077) after accounting for age. Fasting glucose and HbA1c remained significantly related to FSF after accounting for TSI. All relationships were no longer significant after accounting for LOI, with the exception of VAT and TNF-*α*.

Table 4 shows the relationships between FSF and metabolic profile after accounting for VAT, TNF-*α* and IL-6. These relationships remained significant after accounting for VAT, but were no longer significant after accounting for inflammatory cytokines.

## 4. Discussion

Individuals with SCI are at greater risk for several carbohydrate and lipid disorders, such as type II diabetes, metabolic syndrome, and obesity, compared to the general population [1, 3]. Previous studies have shown the link between increased ectopic adipose tissue, inflammation, and metabolic dysfunction in able-bodied individuals [18]. Despite this, few studies have investigated liver adiposity as a risk factor for metabolic dysfunction after SCI. In the current study, we quantified FSF noninvasively by MRI in a chronic SCI population. FSF was linked to several metabolic variables independent of age, TSI, and VAT. Moreover, FSF was independently related to VAT and TNF-*α*, suggesting that FSF is linked to inflammation. Liver adiposity may be an independent risk factor for metabolic dysfunction after SCI.

### 4.1. FSF and Lipid and Metabolic Profiles

After passing through the intestines, nutrients including glucose, fatty acids, and amino acids travel through the hepatic portal vein upon delivery to the liver, which regulates metabolic homeostasis [45]. Here, triglycerides are synthesized from glucose during lipogenesis and are transported through the blood and to the muscle by VLDL-C [45]. Increased hepatic lipids may impair the ability of insulin to regulate gluconeogenesis; however, lipogenesis remains unaffected [45, 46]. In the current study, the association between VLDL-C and liver adiposity is exemplified by the positive relationship between FSF and non-HDL-C, which confirms previous reports in able-bodied individuals [47, 48]. Although we did not measure VLDL-C separately from LDL-C, no relationship was observed between FSF and HDL-C or LDL-C, highlighting the importance of VLDL-C related to liver metabolism [49]. Surprisingly, there was no relationship between FSF and FFA in the current study, which is in contrast to previous studies of persons with NAFLD [50]. However, the cumulative effect of VAT and FSF (FSF-VAT index) resulted in a stronger association with FFA in the current study.

FSF was positively related to blood glucose levels and negatively related to insulin sensitivity in the current study, in agreement with previous research studies [47, 51, 52]. The progression of insulin resistance may explain these findings. Insulin resistance inhibits skeletal muscle glucose uptake by impairing glucose transporter 4 (GLUT4) translocation to the membrane [46]. This results in increased blood glucose and delivery back to the liver, where it may be stored as adipose tissue due to de novo lipogenesis. Furthermore, insulin resistance stimulates the actions of hormone sensitive lipase, which is the rate-limiting enzyme for lipolysis [53]. An increase in lipolysis and increased delivery of dietary glucose combined with increased lipogenesis promotes reesterification of lipids in liver and muscle, further exacerbating insulin resistance.

Increased ectopic adipose tissue such as VAT contributes to metabolic dysfunction. More recently, there has been speculation regarding the relative contributions of VAT and liver adiposity to the development of metabolic disease [47, 54]. Previous studies have shown that liver adiposity predicted metabolic dysfunction [55] and insulin resistance [54] independent of VAT. The current study extends these findings to the SCI population. The associations between FSF and metabolic regulation remained significant when VAT was accounted for. Additionally, the cumulative effect of FSF and VAT (FSF-VAT index) resulted in less significant relationships with metabolic profile compared to FSF alone. Collectively, these findings suggest that liver adipose tissue was negatively associated with metabolic profile independently of VAT in the current study. This highlights the importance of preventing and/or managing liver adiposity, in addition to VAT, in order to avert metabolic dysfunction. Liver adiposity may be decreased by weight loss, through exercise and diet, or by pharmacological intervention such as metformin or pioglitazone [44].

Autonomic dysfunction may contribute to decreased hormone release such as testosterone and further exacerbate metabolic dysfunction and liver adiposity after SCI [9, 56]. Low testosterone (<325 ng/dl) has been shown to be linked to metabolic diseases like type II diabetes, metabolic syndrome, and insulin resistance in able-bodied populations [57–60]. Studies using testosterone replacement therapy have found that testosterone increases lean mass, reduces fat mass, and improves metabolic profile [61, 62]. Twenty percent of the participants in the current study had low testosterone. Interestingly, this inverse association between FSF and testosterone was independent of VAT. Similarly, Barbonetti et al. found that the risk for NAFLD increased for every 1 ng/dl decline in testosterone in individuals with SCI [30].

### 4.2. FSF, Trunk Adipose Tissue, and Inflammation

Based on previous findings, eighty-two percent of participants in the current study are obese based on VAT cutoff > 100 cm^2^ [63] despite having a normal BMI. This cutoff was established by using DXA and CT scan measurements of VAT in persons with SCI [63]. We have previously shown that individuals with chronic SCI who have VAT CSA > 100 cm^2^ are likely to experience insulin resistance [10]. Previous studies have shown that VAT positively correlates with liver adiposity and that these two sources of ectopic adipose tissue have a negative effect on insulin sensitivity [17, 47, 64]. The current study extends these findings to individuals with SCI. The mechanisms underlying this process remain unclear and may include signaling by lipid intermediates (e.g., diacylglycerol and ceramides) and increased inflammation [46]. One hypothesis for the link between VAT, liver adipose tissue, and metabolic dysfunction is that release of inflammatory cytokines such as TNF-*α* and IL-6 from activated macrophages invades ectopic adipose tissue [65, 66] and causes prolonged metabolic stress. TNF-*α* has been shown to inhibit insulin signaling via insulin receptor substrate 1 (IRS1) and may increase adipose lipolysis [46]. Previous studies have found that liver adiposity and inflammation were positively related, which supports this hypothesis [17, 67, 68]. The current study found an independent positive relationship between FSF and TNF-*α*. VAT was also strongly associated with TNF-*α*. It is interesting to note that the strong associations between liver adiposity and metabolic profile were no longer significant after accounting for TNF-*α*, which may support the hypothesis that liver adiposity exerts its negative effects on metabolism via inflammatory cytokines. Future studies may want to further investigate the mechanisms underlying the relationship between liver adiposity and inflammation in a SCI population.

## 5. Limitations

The study criteria excluded individuals with uncontrolled diabetes or known cardiovascular disease [41]. Because the study intervention included testosterone replacement therapy, only men were included. For these reasons, the current findings may not be generalizable to the entire SCI population; however, individuals with motor complete SCI represent ~45% of the SCI population [69]. Because the study was not powered to investigate liver adiposity, confounding variables were not accounted for during MRI acquisition. Furthermore, the use of MRI is limited to clinical settings and requires significant funding for research use, which may make the current findings difficult to replicate in a large cohort of individuals with SCI. While this data should be interpreted with caution, it may be used to drive future research hypotheses. Future studies may warrant the use of a larger sample of both men and women with a range of BMI as well as those with metabolic disorders.

## 6. Conclusions

Liver adiposity was quantified noninvasively in persons with chronic SCI. Increased FSF was associated with lipid and metabolic variables such as increased TG, non-HDL-C, fasting glucose, and HbA1c and decreased *S*_i_ and testosterone. Increased FSF was also associated with increased VAT and TNF-*α*, which was independent of age, LOI, and TSI. Associations between FSF and metabolic profile were independent of VAT but not inflammation, highlighting that inflammation may be the mechanism by which liver adiposity negatively impacts metabolic profile in this population. This highlights the importance of quantifying liver adiposity after SCI in order to prevent the development of NAFLD.

## Figures and Tables

**Table 1:** Physical characteristics of persons with motor complete SCI.

	Total, *n* = 22	Tetra, *n* = 8	Para, *n* = 14
Demographics			
Age, year	36.1 ± 10.0	37.5 ± 11.6	35.3 ± 9.4
Height, m	1.79 ± 0.06	1.80 ± 0.05	1.78 ± 0.07
Weight, kg	78.0 ± 13.0	75.0 ± 14.0	80.0 ± 13.0
BMI, kg/m^2^	24.6 ± 3.9	23.3 ± 4.5	25.3 ± 3.4
TSI, y	8.2 ± 7.9	7.9 ± 7.2	8.4 ± 8.5
Caucasian,* n*	14	6	8
African American, *n*	8	2	6

Values are means ± SD; *n*, number of participants; Tetra, tetraplegic; Para, paraplegic; BMI, body mass index; TSI, time since injury.

**Table 2:** Lipid profile, glucose profile, ectopic adipose tissue, and inflammation in persons with motor complete SCI.

	Total, *n* = 22	Tetra, *n* = 8	Para,* n* = 14
Lipid profile			
Total cholesterol (mg/dl)	150.1 ± 29.0	160.4 ± 21.1	144.3 ± 31.9
TG (mg/dl)	111.1 ± 54.5	141.1 ± 68.2	93.9 ± 37.6
LDL-C (mg/dl)	92.6 ± 26.9	98.4 ± 23.2	89.3 ± 29.1
HDL-C (mg/dl)	35.0 ± 8.1	33.0 ± 6.5	36.1 ± 8.9
Non-HDL-C (mg/dl)	111.9 ± 32.3	127.4 ± 16.7	103.1 ± 36.2^*∗*^
Total cholesterol: HDL-C	4.4 ± 1.0	4.9 ± 0.7	4.1 ± 1.0^*∗*^
FFA (*µ*M/L)	363.7 ± 188.8	394.1 ± 216.6	346.3 ± 177.2
Metabolic profile			
Fasting glucose (mg/dl)	107.1 ± 13.0, *n* = 21	114.5 ± 14.54	102.5 ± 9.8, *n* =13^∧^
*S*_i_ ((mU/L)^−1^ min^−1^)	8.62 ± 6.28, *n* = 19	4.61 ± 6.07, *n* = 6	10.47 ± 5.67, *n* = 13^∧^
*S*_g_ (min^−1^)	0.020 ± 0.009	0.017 ± 0.003	0.022 ± 0.011
HbA1c (%)	5.35 ± 0.45, *n* = 21	5.61 ± 0.50	5.19 ± 0.34, *n* = 13^∧^
Testosterone (ng/dl)	424.4 ± 159.1	345.3 ± 125.5	469.6 ± 162.3^∧^
SAT and VAT			
Trunk CSA (cm^2^)	654.4 ± 139.6	664.7 ± 166.8	648.5 ± 128.0
SAT (cm^2^)	157.0 ± 79.6	163.5 ± 103.2	153.4 ± 66.7
VAT (cm^2^)	105.4 ± 63.9	117.5 ± 57.2	98.4 ± 68.6
Inflammation			
TNF*α* (pg/ml)	15.3 ± 8.0	16.0 ± 8.2	14.9 ± 8.1
IL-6 (pg/ml)	5.89 ± 6.77	5.03 ± 5.39	6.39 ± 7.59
Liver analysis (*n*)	20	7	13
FSF (%)	3.73 ± 2.10	5.34 ± 2.05	2.86 ± 1.59^*∗*^
Liver CSA (cm^2^)	163.4 ± 31.8	158.0 ± 30.5	166.3 ± 33.4

Values are means ± SD; *n*, number of participants; Tetra, tetraplegic; Para, paraplegic; LDL, low-density lipoprotein cholesterol; HDL-C, high-density lipoprotein cholesterol; FFA, free-fatty acids; *S*_i_, insulin sensitivity; *S*_g_, glucose effectiveness; HbA1c, glycated hemoglobin; SAT, subcutaneous adipose tissue; VAT, visceral adipose tissue; TNF*α*, tumor necrosis factor alpha; IL-6, interleukin-6; FSF, fat signal fraction; CSA, cross-sectional area; ^∧^*p* ≤ 0.1, ^*∗*^*p* < 0.05 tetra versus para.

**Table 3:** Relationships and partial correlations between FSF, lipid panel, glucose profile, and inflammation accounting for age, level of injury (LOI), and time since injury (TSI).

	Pearson	Partial (age)	Partial (LOI)	Partial (TSI)
Non-HDL-C (mg/dl)	0.503^*∗*^	0.350^*∗*^	0.366	0.509
TG (mg/dl)	0.538^*∗*^	0.515^*∗*^	0.378	0.720^*∗∗*^
*S*_i_ ((mU/L)^−1^ min^−1^)	−0.605^*∗*^	−0.553^*∗*^	−0.423	−0.618^*∗*^
Fasting glucose (mg/dl)	0.513^*∗*^	0.478^∧^	0.380	0.518^*∗*^
HbA1c (%)	0.506^*∗*^	0.470^∧^	0.370	0.511^*∗*^
Testosterone (ng/dl)	−0.466^*∗*^	−0.475^∧^	−0.345	−0.467^∧^
VAT (cm^2^)	0.480^*∗*^	0.531^*∗*^	0.489^*∗*^	0.555^*∗*^
TNF-*α* (pg/ml)	0.598^*∗*^	0.531^*∗*^	0.629^*∗*^	0.617^*∗*^

HDL-C; high-density lipoprotein cholesterol; TG, triglycerides; *S*_i_, insulin sensitivity; HbA1c, glycated hemoglobin; VAT, visceral adipose tissue; TNF-*α*, tumor necrosis factor alpha. ^∧^*p* ≤ 0.1, ^*∗*^*p* < 0.05, and ^*∗∗*^*p* < 0.01.

**Table 4:** Relationships and partial correlations between FSF and metabolic profile accounting for VAT and inflammatory cytokines.

	Pearson	Partial (VAT)	Partial (TNF-*α*)	Partial (IL-6)
*S*_i_	−0.605^*∗*^	−0.605^*∗*^	−0.361	0.054
Fasting glucose	0.513^*∗*^	0.502^*∗*^	0.331	−0.378
HbA1c	0.506^*∗*^	0.532^*∗*^	0.312	−0.382
Testosterone	−0.466^*∗*^	−0.553^*∗*^	−0.420	0.063

FSF, fat signal fraction; *S*_i_, insulin sensitivity; HbA1c, glycated hemoglobin; VAT, visceral adipose tissue; TNF-*α*, tumor necrosis factor alpha; IL-6, interleukin-6. ^*∗*^*p* < 0.05.

## Abbreviations

^1^H-MRS: Proton magnetic resonance spectroscopy
ASIA: American spinal cord injury association
AIS: ASIA impairment scale
BMI: Body mass index
CSA: Cross-sectional area
CT: Computed tomography
FFA: Free-fatty acids
FSF: Fat signal fraction
GLUT4: Glucose transporter 4
HbA1c: Glycated hemoglobin
HDL-C: High-density lipoprotein cholesterol
IL-6: Interleukin 6
IRS1: Insulin receptor substrate 1
ISNCSCI: International Standards for Neurological Classification of SCI
IP: In-phase
LOI: Level of injury
LDL-C: Low-density lipoprotein cholesterol
MRI: Magnetic resonance imaging
NAFLD: Nonalcoholic fatty liver disease
OP: Out-of-phase
SAT: Subcutaneous adipose tissue
SCI: Spinal cord injury
SD: Standard deviation
*S*_g_: Glucose effectiveness
*S*_i_: Insulin sensitivity
TG: Triglycerides
TNF-*α*: Tumor necrosis factor alpha
TSI: Time since injury
VAT: Visceral adipose tissue
VLDL-C: Very low-density lipoprotein cholesterol.
